# Characterization of CD30/CD30L^**+**^ Cells in Peripheral Blood and Synovial Fluid of Patients with Rheumatoid Arthritis

**DOI:** 10.1155/2015/729654

**Published:** 2015-05-19

**Authors:** Alessandro Barbieri, Marzia Dolcino, Elisa Tinazzi, Antonella Rigo, Giuseppe Argentino, Giuseppe Patuzzo, Andrea Ottria, Ruggero Beri, Antonio Puccetti, Claudio Lunardi

**Affiliations:** ^1^Department of Medicine, University of Verona, 37134 Verona, Italy; ^2^Institute G. Gaslini, 16147 Genova, Italy; ^3^University of Genova, 16132 Genova, Italy

## Abstract

The CD30/CD30L signalling system has been implicated in the pathogenesis of several autoimmune and inflammatory conditions. In rheumatoid arthritis (RA), soluble CD30 (sCD30) levels reflect the recruitment of CD30^+^ T cells into the inflamed joints and correlate with a positive response to immunosuppressive therapy. The aim of our report was to clarify the role of CD30/CD30L signalling system in the pathogenesis of RA. Our analysis of the CD30L^+^ T cell subsets in peripheral blood (PB) and synovial fluid (SF) of RA patients and of the related cytokine profiles suggests the involvement of CD30/CD30L signalling in polarization of T cells towards a Th17 phenotype with proinflammatory features. Moreover, in RA SF nearly 50% of Treg cells express CD30, probably as an attempt to downmodulate the ongoing inflammation. We also show here that the engagement of CD30L on neutrophils stimulated with CD30/Fc chimera may play a crucial role in RA inflammation since activated neutrophils release IL-8, thus potentially amplifying the local inflammatory damage. In conclusion, the results obtained suggest that the complex CD30/CD30L signalling pathway is implicated in the pathogenesis and progression of RA synovitis through a concerted action on several immune effector cells.

## 1. Introduction

CD30 is a member of the tumour necrosis factor receptor (TNFR) superfamily that includes, among others, TNFR, CD40, Fas (CD95), and OX-40 (CD134) [1]. Human CD30 is a type 1 glycoprotein and its cytoplasmic region is characterized by the presence of several serine/threonine phosphorylation sites which regulate cell signalling, once the receptor is engaged [2]. In nonpathological conditions, activated T- and B-lymphocytes and NK cells generally express the CD30 molecule, while lower levels of expression are present in activated monocytes and eosinophils [3]. Moreover, CD30 is found on a small percentage of CD8^+^ T cells while only a negligible expression on naïve or resting lymphocytes has been described [4]. Several mechanisms are able to trigger CD30 expression on T cells, including mitogen activation, antigen receptor cross-linking, and viral infections [5].

CD30L belongs to the TNF family [2] and is the only known ligand for CD30. It is found, at RNA transcription level, in B cells, activated T cells, macrophages, granulocytes, eosinophils, and some HTLV-1-positive T cell lines [6–13], while at protein level, CD30L is detected on activated peripheral blood T cells, B cells, neutrophils, mast cells, monocytes, and macrophages [14]. The CD30L molecule is cleaved and released by CD30L^+^ cells in presence of CD30^+^ cells and the soluble form of CD30L (sCD30L) has the ability to induce CD30^+^ cells apoptosis [15].

Interaction of CD30L with cells expressing CD30 induces signals that cause cell proliferation or cell death. Interestingly, upon binding to CD30, CD30L is also able to transduce a signal. One of the possible consequences of this reverse signalling is cell proliferation [12].

The role of CD30/CD30L interaction in health and disease is still not totally understood, in part due to the pleiotropic nature of CD30 signals.

*In vitro*, CD30 expression has been mainly associated with a Th2/Th0 phenotype [16], although* in vivo* studies suggest that the relationship between CD30^+^ T cells and Th1 or Th2 profiles is very complex. Some of us have proposed a novel regulatory mechanism for CD30 in Th1 polarized responses such as rheumatoid arthritis [17].

Indeed in autoimmune and chronic inflammatory diseases, several studies have provided evidences that CD30/CD30L signalling is involved in T helper (Th)2 cell responses and Th2-associated diseases [18, 19]. However, recent observations have shown that CD30/CD30L signalling plays a role also in Th1 and Th17 responses and in Th1-associated diseases [16, 20–22]. Furthermore, it is also involved in the regulation of memory T-cell response: in a murine transplantation model, antigen-induced T regulatory (Treg) cells, but not naïve ones, were able to suppress allograft rejection mediated by memory CD8^+^ T cells in an Ag-specific manner [23]. This suppression was related to an enhanced apoptosis of allospecific memory CD8^+^ T cells in the graft due to the presence of CD30 expressing regulatory T (Treg) cells and to the CD30/CD30L interaction [24–26].

As far as rheumatoid arthritis (RA) concerns, patients affected by the disease have increased levels of soluble CD30 (sCD30) in both serum and synovial fluid (SF) [27]. This feature could reflect the presence of CD30^+^ synovial T lymphocytes, recruited at the site of tissue aggression with the possible aim to downmodulate inflammation [28].

These data may be crucial for the understanding of the cellular mechanisms underlying clinical response to classical and biological disease modifying drugs since we have shown that sCD30 levels correlate with response to treatment [29, 30], whereas high levels of sCD30L seem to predict lack of response to biological therapy [15].

The complex CD30/CD30L signalling pathway is indeed further complicated by the role played by the soluble forms of CD30 and CD30L. In animal model sCD30 is able to inhibit CD30/CD30L interaction and, at the same time, activate CD30L by reverse signalling [20]. Moreover, in a murine model, inhibition of CD30/CD30L signalling by viral encoded CD30 leads to a decrease in Th1 cytokines production, such as for IFN-*γ*, conferring to this mechanism a potential relevant role in the control of a Th1-response to viral infection [20].

RA is considered primarily a Th1-driven condition [31, 32] although the presence of increased levels of Th2 cytokines, such as IL-4 and IL-10, is found in the early stages of the disease [27]. It has been reported that IL-4 has the ability to induce CD30 membrane expression [33] and increased levels of IL-4 have been found in both SF and serum of RA patients [34].

CD30/CD30L signalling is also involved in Th17 induction. CD4^+^ T cells taken from CD30L- or CD30-deficient mice showed a reduced ability to differentiate into Th17 cells [31].* In vivo* experiments showed that transfection of CD30L^−/−^ CD4^+^ T cells in severe combined immunodeficiency (SCID) CD30L-deficient mice leads to an altered Th17 differentiation, while transferring CD30L^+/+^ CD4^+^ T cells causes a normal Th17 differentiation. The data suggest that CD30/CD30L signalling carried out by the T-T cell interaction plays a critical role in Th17 cell differentiation [21, 35, 36].

Therefore the CD30/CD30L signalling has different effects depending on the disease and on the cytokine milieu in which it takes place. Moreover it is further complicated by the functional activity of the soluble molecules.

The aim of this work was to clarify some of the controversial issues related to the role played by CD30/CD30L^+^ cells in the pathogenesis of rheumatoid synovitis.

## 2. Materials and Methods

### 2.1. Patients

Fourteen patients (10 females and 4 males, mean age 54 ± 16) affected by RA were enrolled in the study. RA patients met the American College of Rheumatology classification criteria for RA [37]. Eight donors with posttraumatic synovitis were used as controls.

A written informed consent was obtained from all of the participants in the study. The study was approved by local Ethical Committee. All clinical investigations have been conducted according to the principles expressed in the Helsinki declaration.

### 2.2. Isolation of Peripheral Blood and Synovial Fluid Mononuclear Cells

Peripheral blood mononuclear cells (PBMCs) and synovial fluid mononuclear cells (SFMCs) were isolated from heparinized PB and from SF treated with hyaluronidase (Sigma, St. Louis, MO) through density gradient centrifugation using Lymphoprep Ficoll-Isopaque (Axis-Shield, Oslo, NO) according to manufacturer's instruction. Briefly, 10 mL PB or SF was diluted with 10 mL PBS and then layered over 10 mL Lymphoprep in a 50 mL centrifuge tube. Samples were centrifuged at 800 ×g for 20 minutes and cells were collected using a Pasteur pipette. Cells were washed twice with PBS at 1200 rpm for 10 minutes and then counted in a Burker chamber using acridine orange staining.

### 2.3. PBMCs and SFMCs Immunophenotype by FACS Analysis

The immunophenotypic analysis of PBMC and SFMC populations was carried out using flow cytometry. From each sample we obtained three different tubes containing 1 × 10^6^ cells each that were stained with different monoclonal antibodies. Samples for CD30 detection were stained with CD4 APC-H7 (5 *µ*L), CD8 FITC (10 *µ*L), CD3 PerCp (10 *µ*L), and CD30 PE antibodies (10 *µ*L); samples for CD30L detection were stained with CD4 APC-H7 (5 *µ*L), CD8 FITC (10 *µ*L), CD3 PerCp (10 *µ*L), and CD30L PE antibodies (20 *µ*L); finally samples for Treg cells detection were stained with CD4 APC-H7 (5 *µ*L), CD25 FITC (20 *µ*L), CD3 PerCp (10 *µ*L), CD127 PE-Cy7 (5 *µ*L), and CD30 PE antibodies (10 *µ*L). Staining of surface molecules was performed for 20 minutes at room temperature protected from the light. Treg tubes were then fixed, permeabilized, and incubated with ALEXA647 FoxP3 antibody. Briefly, cells were washed with 2 mL wash buffer at 250 ×g for 10 minutes and then fixed with Human Foxp3 Buffer A for 10 minutes at room temperature. Cells were washed and then permeabilized incubating with 0.5 mL working solution Human Foxp3 Buffer C for 30 minutes. Cells were then washed and stained with FoxP3 ALEXA647 monoclonal antibody (20 *µ*L) for 30 minutes. All reagents were purchased from Becton Dickinson (San Jose, CA, USA), except for CD30L monoclonal antibody (R&D System, Minneapolis, MN, USA). Samples were analysed on a FACSCanto cytometer (Becton Dickinson). Data were analysed by FlowJo 9.3.3 software (Tree Star, Ashland, OR).

### 2.4. Time Course of CD30 and CD30L Cell Surface Expression

PBMCs from healthy donors were activated with Dynabeads Human T-Activator CD3/CD28 (Life Technologies) in a 14 mL polypropylene round-bottom tube with a ratio of 1 : 1 and incubated for 6, 10, 15, 20, 24, and 48 hours in RPMI + 10% FCS at 5% CO_2_. Cells were harvested, washed, and stained for 20 minutes with CD3 APC (Becton Dickinson) and CD30 PE (Becton Dickinson) or CD30L PE (R&D System) antibodies. Cells were acquired on a FACSCalibur cytometer (Becton Dickinson) and analysed with FlowJo 9.3.3 software (Tree Star).

### 2.5. PBMCs and SFMCs Stimulation by CD30/Fc Chimera

PBMCs and SFMCs were activated as described above and cultured in presence or absence of 20 *µ*g/mL CD30/Fc chimera (R&D Systems). PBMCs were harvested after 24, 48, and 120 hours of incubation, while SFMCs were harvested after 15, 24, and 48 hours. Cells and supernatants were collected for RNA extraction and cytokine levels evaluation, respectively.

### 2.6. RNA Isolation and RT-PCR

Total RNA was extracted from PBMCs using TRIzol reagent (Invitrogen, Carlsbad, CA, USA), following manufacturer's instructions. First-strand cDNA was generated using the SuperScript III First-Strand Synthesis System for RT-PCR Kit (Invitrogen), with random hexamers, according to the manufacturer's protocol. cDNA was aliquoted in equal volumes and stored at −20°C.

### 2.7. Cytokines Production Evaluated by Real Time RT-PCR

PCR was performed in a total volume of 25 *μ*L containing 1× TaqMan Universal PCR Master mix, no AmpErase UNG, and 2.5 *μ*L of cDNA; predesigned, gene-specific primers and probe sets for each gene (IL-17 Hs00174383_m1, IL-4 Hs00174122_m1, IL-6 Hs00985639_m1, IFN-*γ* Hs00989291_m1, and IL-10 Hs00961622_m1) were obtained from Assay-on-Demand Gene Expression Products (Applied Biosystems, Foster City, CA, USA). Real Time PCR reactions were carried out in a two-tube system and in singleplex. The real time amplifications included 10 minutes at 95°C (AmpliTaq Gold activation), followed by 40 cycles at 95°C for 15 seconds and at 60°C for one minute. Thermocycling and signal detection were performed with 7500 Sequence Detector (Applied Biosystems). Signals were detected according to the manufacturer's instructions. This technique allows the identification of the cycling point where PCR product is detectable by means of fluorescence emission (threshold cycle or Ct value). The Ct value correlates with the starting quantity of target mRNA. Relative expression levels were calculated for each sample after normalization against the housekeeping gene GAPDH, using the ΔΔCt method for comparing relative fold expression differences. The data are expressed as fold change. A fold change >1.5 was considered a significant increase in transcription. Ct values for each reaction were determined using TaqMan SDS analysis software. For each amount of RNA tested triplicate Ct values were averaged. Because Ct values vary linearly with the logarithm of the amount of RNA, this average represents a geometric mean.

### 2.8. Cytokines Secretion Evaluated by ELISA Kit

Supernatants of the previously described experiment were tested for cytokine levels using commercial ELISA kit purchased from R&D Systems (Human Quantikine ELISA). IL-6, IL-10, IL-4, IFN-*γ*, and IL-17 were evaluated in the supernatants following the manufacturer instructions. Plates were read at 450 nm with TECAN Sunrise III (Tecan).

### 2.9. Cytokines Secretion Assay by Flow Cytometry

PBMCs were activated for 24 hours with Dynabeads Human T-Activator CD3/CD28 (Life Technologies) and cultured for 3 hours with or without 20 *µ*g/mL CD30/Fc chimera (R&D Systems) in RPMI + 10% FCS. Cells were then harvested and tested for IL-17 secretion using Cytokine Secretion Assays (Miltenyi Biotec, Bergisch Gladbach, Germany) following manufacturer's instructions. Briefly, cells were suspended in 2 mL medium, washed with 2 mL cold buffer at 300 ×g for 10 minutes, and resuspended in 90 *μ*L cold medium. Cells were incubated 5 minutes on ice with 10 *μ*L IL-17 catch reagent. Cells were diluted with warm medium and incubated for 45 minutes at 37°C, washed with cold buffer and then stained with CD3 PerCp (Becton Dickinson), CD30L PE (R&D Systems), and IL-17 Detection Antibody APC. Cells were acquired on a FACSCanto cytometer (Becton Dickinson). Analysis was performed with FlowJo 9.3.3 software (Tree Star).

### 2.10. Neutrophils Isolation

Neutrophils from fresh buffy coat of normal subjects were isolated through density gradient centrifugation using Lymphoprep Ficoll-Isopaque (Axis-Shield, Oslo, NO) according to manufacturer's instruction. Briefly, 10 mL of PB was diluted with 10 mL PBS and then layered over 10 mL Lymphoprep in a 50 mL centrifuge tube. Samples were centrifuged at 800 ×g for 20 minutes and cells were collected using a Pasteur pipette. Obtained cells were put in a new tube in a final volume of 40 mL PBS. In order to achieve erythrocytes sedimentation, 8 mL of dextran was added and after 10–15 minutes, after separation of phases, supernatant was transferred in a new tube. Cells were washed with PBS at 1200 rpm for 5 minutes and 7.5 mL of NaCl 0.2% was added. After 50 seconds, 17.5 mL of NaCl 1.2% was added; cells were mixed gently and washed again with PBS. Cells were then counted using acridine orange staining in a Burker chamber and purified using the EasySep Negative Selection Human Neutrophil Enrichment Kit (StemCell Technologies, Vancouver, Canada) according to manufacturer's instructions. Briefly, cells were placed in a 5 mL polystyrene tube at a concentration of 50 × 10^6^ cells/mL and incubated for 10 minutes with 100 *μ*L EasySep Human Neutrophil Enrichment Cocktail. Two hundreds microL EasySep Nanoparticles were then added into the tube and after 10 minutes the tube was placed in a magnet for 5 minutes. Magnet and tube were inverted and the desired fraction was collected in a new tube. Neutrophils were counted as described above.

### 2.11. CD30L Expression on Neutrophils

RNA isolation and transcription have already been described. The amplified DNA obtained after RT-PCR was run in a 1.5% agarose gel. cDNA obtained from DG75 cells (human Burkitt lymphoma, DSMZ) that constitutively express CD30L was used as positive control. In order to analyse CD30L membrane expression, purified neutrophils were also preincubated with normal human serum (Invitrogen) for 10 minutes and then incubated with CD30L PE monoclonal antibody (R&D Systems) for 20 minutes. Cells were acquired on a FACSCanto cytometer (Becton Dickinson) and analysis was performed with FlowJo 9.3.3 software (Tree Star).

### 2.12. Neutrophils Cytokines Secretion Evaluated by ELISA Kit

Neutrophils were seeded in a 6-well plate at a final concentration of 2 × 10^6^ cells/mL in RPMI + 10% FCS. They were cultured alone and with 20 *μ*g/mL CD30/Fc chimera (R&D Systems), with 10 ng/mL LPS (Invitrogen), or with both CD30/Fc chimera and LPS as a costimulus. Cells were harvested after 1, 3, and 10 hours and levels of IL-8, TNF-*α*, MMP-9, VEGF, IFN-*γ*, and IL-17 were evaluated using commercial ELISA kits purchased from R&D Systems (Human Quantikine ELISA). Plates were read at 450 nm with TECAN Sunrise III (Tecan).

### 2.13. Statistical Analysis

All the calculations were performed with SPSS 21.0 statistical package (SPSS Inc., Chicago, IL, USA). Quantitative data with a normal distribution are expressed as mean ± SD and were analysed with Student's *t*-test.

A value of *P* < 0.05 was considered significant.

## 3. Results and Discussion

### 3.1. Immunophenotypic Analysis of T Cells from Patients with RA

In order to better clarify the expression of CD30 and CD30L molecules by T cells in RA we analysed the expression of these molecules by CD4^+^ and CD8^+^ T cell subsets in both PB and SF of 14 RA patients and 8 control subjects. In the same samples we also evaluated the frequency of Treg cells expressing the CD30 molecule. Mean values of percentages of each subpopulation in RA patients and control subjects are reported in Table 1.

In PB samples, we did not find any significant difference in the percentages of all CD30^+^ T cell subsets between RA patient and control groups. On the contrary, in SF samples, we found that RA patients had a percentage of CD4^+^ T cells expressing the CD30 molecule much higher than in controls (8.6% ± 5.6 versus 0.5% ± 0.7).

The percentage of Treg population among total CD4^+^ cells in the SF of RA patients was higher than the percentage found in the SF of control subjects (13.8% ± 4.8 versus 4.0% ± 2.8). Remarkably in RA patients 42.3% ± 14.7 of the total Treg cells also express the CD30 molecule whereas only 19.0% ± 11.3 of the total Treg subset are CD30^+^ in the control subjects (Table 1).

Such a high frequency of Treg cells may indicate that in patients affected by RA synovitis there is an attempt to downmodulate inflammation in order to control disease progression. Moreover, the high percentage of Treg expressing the CD30 molecule strengthens the knowledge that CD30 is expressed by cells that have an anti-inflammatory behaviour.

Finally, we did not observe differences in CD30L^+^ T cell percentages in the different T-cell subsets between RA patients and the control group. The only significant difference is represented by the percentage of CD4^+^CD30L^+^ T cell subset that is higher in SF than in PB samples in both patients and controls, suggesting that CD30L molecule is preferentially expressed by cells present at sites of inflammation.

### 3.2. CD30 and CD30L Expression over Time upon Cell Activation

The expression of surface CD30 and CD30L molecules is induced on T cells following activation with CD3/CD28 beads. In order to study the kinetics of expression of surface CD30 or CD30L molecule, we performed a time course experiment evaluating the percentage of positive activated T cells from healthy donors.

**Figure 1:**
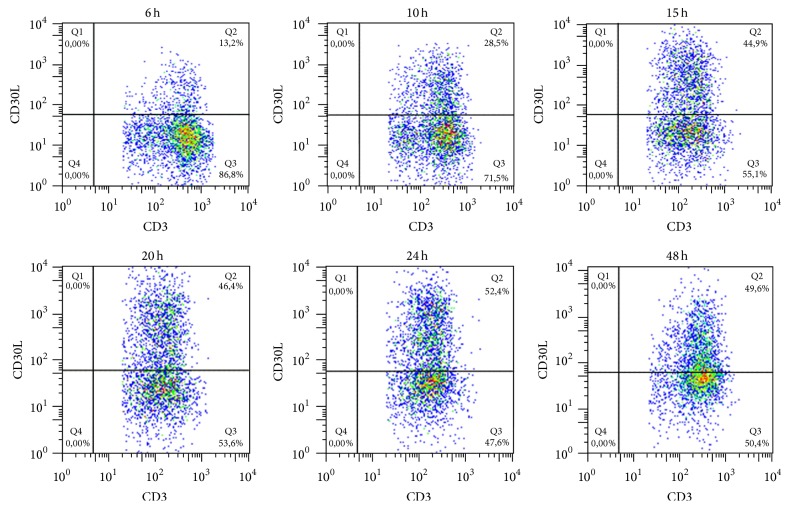
Surface CD30L expression upon cell activation. Percentage of CD30L^+^ T cells rapidly increases in the first 15 hours reaching the maximum value (52.4%) after 24 hours from activation with CD3/CD28 beads.

**Figure 2:**
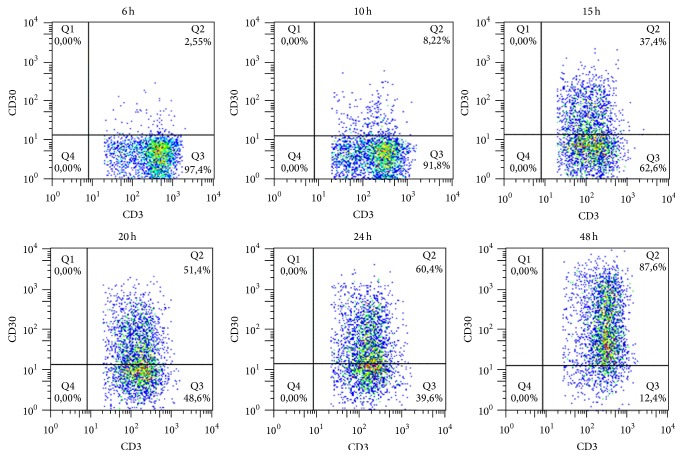
Surface CD30 expression upon cell activation. Percentage of CD30^+^ T cells increases until it reaches the maximum value (87.6%) after 48 hours from activation with CD3/CD28 beads.

We observed an increase in percentage of CD30L^+^ cell population after 15 hours of stimulation with a peak at 24 hours when 52.4% of T cells expressed CD30L on their surface (Figure 1). CD30 expression appears later, since 87.6% of T cells are CD30^+^ 48 hours after stimulation (Figure 2).

Since CD30 is mainly expressed by T cells with a Th2 [16, 17] phenotype and CD30L by T cells with a Th1 phenotype [18–21], the results of this experiment suggest that, upon activation, T cells expressing the CD30L molecule with a proinflammatory profile are mainly present at the first stages of inflammation, whereas cells expressing the CD30 molecule are present later on with the aim to downmodulate and possibly switch off inflammation.

### 3.3. Cytokines Expression Induced by CD30/Fc Chimera

**Figure 3:**
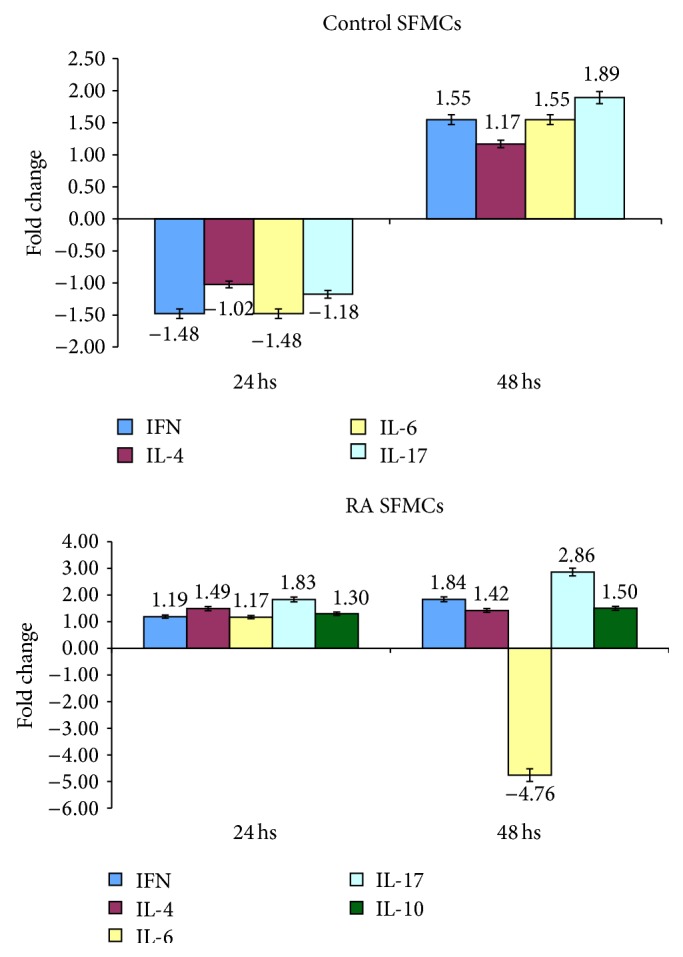
Modulation of expression of genes encoding for cytokines in activated SFMCs following incubation with CD30/Fc chimera in controls and patients with RA.

In order to evaluate the effects of CD30/CD30L signalling in CD30L^+^ T cells we studied the expression of gene transcripts of different cytokines by activated SFMCs from 4 control subjects and from 5 RA patients at 24 and 48 hours. SFMCs from control subjects showed an overexpression of transcripts encoding for IFN-*γ*, IL-6, and IL-17 at 48 hours, whereas SFMCs from patients with RA had an increase of transcripts encoding for IL-17 at 24 and 48 hours and for IFN-*γ* and IL-10 at 48 hours after incubation with CD30/Fc chimera. On the contrary we observed a decrease of gene transcripts for IL-6 after 48 hours (Figure 3).

**Figure 4:**
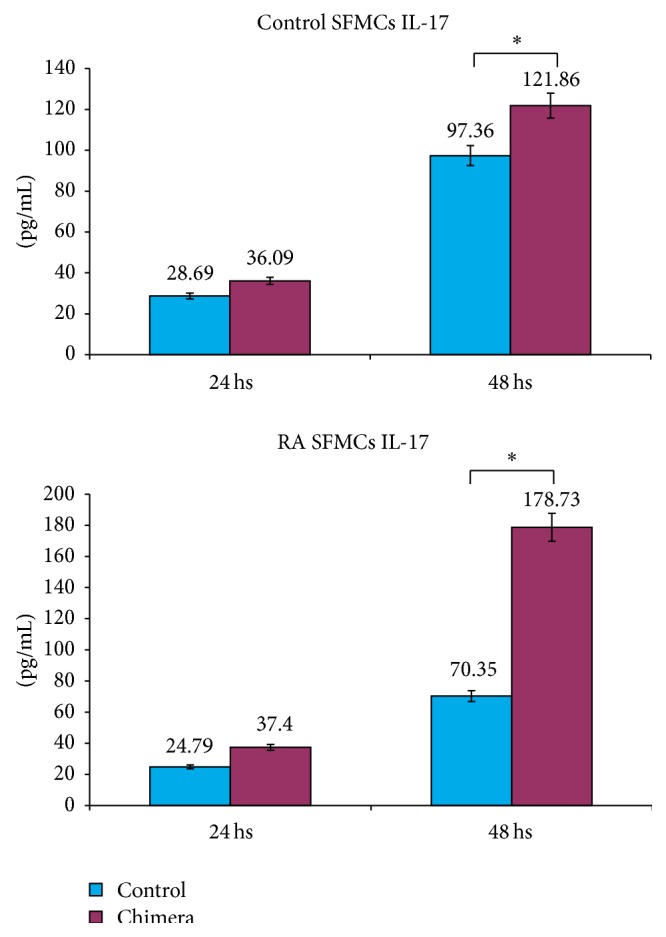
Levels of IL-17 released in the medium by activated SFMCs in presence or absence of CD30/Fc chimera in control subjects and in patients with RA. CD30/Fc chimera stimulus induces the production of IL-17 at 48 hours. ^*∗*^ indicates a* P* value < 0.05.

We then evaluated the levels of IL-17 in the supernatants of SFMCs obtained from control subjects and RA patients 24 and 48 hours after stimulation with the CD30/Fc chimera and found a statistically increased level of the cytokine in the supernatant of SFMCs from both groups at 48 hours after stimulation with the CD30/Fc chimera versus unstimulated samples (Figure 4). On the contrary we did not observe any difference in the detection of the other cytokines studied in the supernatant: IL-6, IL-10, IL-4, and IFN-*γ*.

These data are consistent with the increased transcripts for IL-17 detected by quantitative PCR and indicate the stimulation of CD30L induces the production of a proinflammatory cytokine.

**Figure 5:**
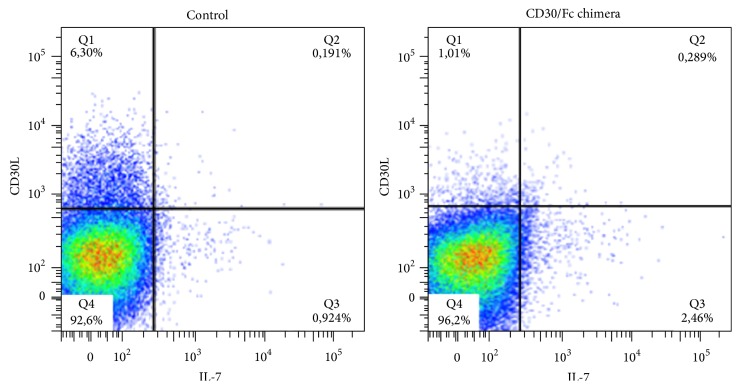
FACS analysis of IL-17 producing cells among SFMCs from RA patients, stimulated with CD30/Fc chimera. After 3 hours of CD30/Fc chimera stimulation, the percentage of IL-17 producing T cell is 2.749%, while it is 1.115% in the unstimulated cells.

Finally, we have evaluated the percentage of IL-17 producing T cells by FACS analysis of cytokine secreting cells using SFMCs obtained from RA patients. We observed that samples cultured in presence of CD30/Fc chimera doubled the percentage of IL-17 producing cells (Figure 5).

Also these data suggest that the stimulation of CD30L^+^ T cells via CD30/Fc chimera promotes the production of IL-17.

The results obtained so far are consistent with an increase of IL-17 (at both transcript and protein levels) suggesting that the CD30/CD30L signalling is involved in polarizing the Th17 response with proinflammatory effects, as already observed in the murine model [20, 35, 36].

It is interesting to note that IL-17 transcripts and supernatant levels are higher in patients with RA compared to controls indicating that SFMCs from patients are more susceptible to IL-17 production.

### 3.4. Neutrophils Express CD30L on Their Surface

**Figure 6:**
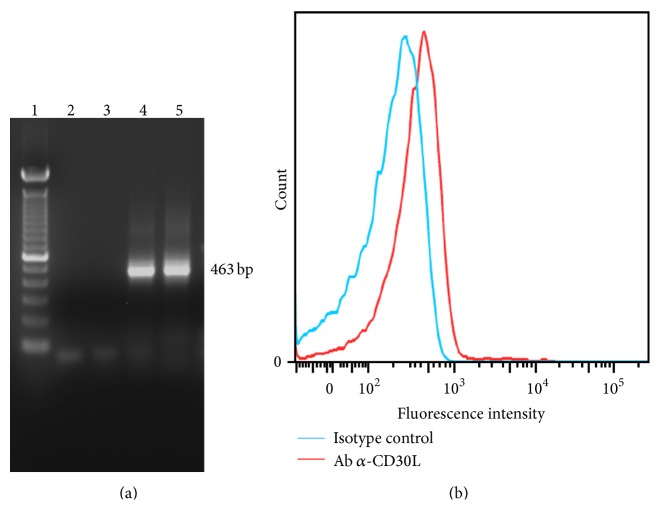
Neutrophils express CD30L. (a) Gel electrophoresis of RT-PCR products obtained from CD30L transcripts in neutrophils (lane 4) and DG75, used as positive control (lane 5) and negative controls (lanes 2 and 3, resp.). (b) CD30L surface expression obtained with FACS analysis.

Neutrophils are thought to play a pivotal role in the pathogenesis of RA inflammation and are also involved in bone erosion, a typical feature of the disease. The presence of CD30L on neutrophils has been investigated long time ago only in one report using a monoclonal antibody which is no more commercially available [12]. For this reason we wanted to confirm this observation at both transcript and protein levels. RT-PCR showed a band at 463 bp for both DG75 (positive control) and neutrophils (Figure 6(a)) and FACS analysis revealed the expression of CD30L on neutrophils cell surface (Figure 6(b)).

### 3.5. Molecules Secreted by Neutrophils Stimulated by CD30/Fc Chimera

**Figure 7:**
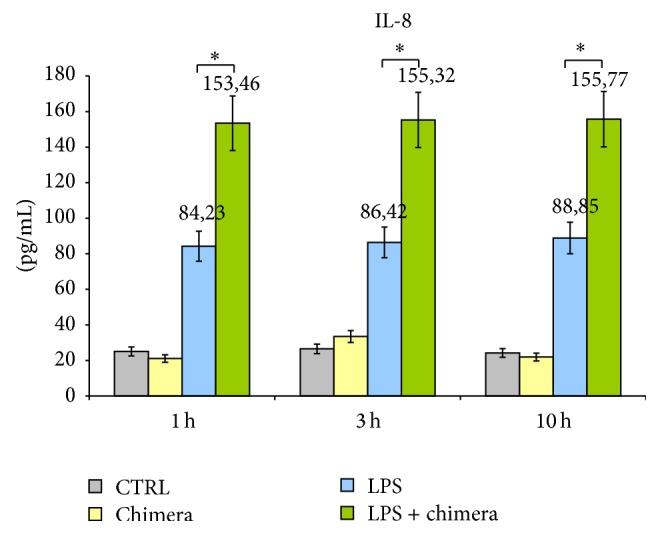
Levels of IL-8 released in the medium by healthy donor's neutrophils in presence or absence of CD30/Fc chimera and activated or not with LPS. Neutrophils incubated with LPS and with CD30/Fc chimera displayed a statistical significant increase in IL-8 production. ^*∗*^ indicates a* P* value < 0.05.

Neutrophils incubated with LPS plus CD30/Fc chimera showed a statistically significant increased level of IL-8 in the culture medium compared with the level detected in the supernatant of neutrophils incubated with CD30/Fc chimera or with LPS alone (Figure 7). The other analysed molecules (TNF-*α*, MMP-9, VEGF, IFN-*γ*, and IL-17) did not show any significant difference with or without CD30/Fc chimera (data not shown).

In conclusion, neutrophils express CD30L and once activated by LPS, in the presence of CD30/Fc chimera, they increase the production of the proinflammatory cytokine IL-8, able to recruit neutrophils at site of inflammation and to stimulate angiogenesis.

## 4. Conclusion

The aim of this study was to clarify the role of CD30/CD30L signalling in the pathogenesis of rheumatoid arthritis. First of all we observed an increased percentage of CD4^+^CD30^+^ T cells in the SF of patients with RA compared with controls suggesting an attempt to control inflammation. This result is in accordance with our previously reported hypothesis on the role of CD30^+^ T cells in RA [18].

Moreover the high percentage of regulatory T cells that display CD30 molecule in the SF of patients affected by RA strengthens the idea that CD30 molecule is expressed by T cells aiming at downmodulating the inflammatory process.

The finding of a higher percentage of CD30L^+^ T cells in SF than in PB suggests that these cells may be involved in favouring local inflammation.

The kinetic of surface expression of CD30 and CD30L molecules seems to further confirm the observations reported above, since we observed a larger population of CD30L^+^ T cells with proinflammatory properties in the first 24 hours after cell activation, whereas a larger CD30^+^ T cell population is present after 48 hours of cell activation suggesting an attempt to switch the inflammatory process off.

We have demonstrated that stimulation of CD30L with the CD30/Fc chimera, a molecule that behaves as sCD30, is able to favor the polarization of T cells towards a Th17 phenotype with proinflammatory features. Indeed this has been shown firstly at gene transcripts level by real time PCR, secondly by evaluating the IL-17 secretion in the supernatant, and finally by analysing the cytokine secretion cells by FACS analysis.

These findings are in accordance with data obtained in animal models [21, 35, 36].

In the last part of this work we have confirmed the presence of surface CD30L on neutrophils and that activated neutrophils release IL-8 once stimulated with CD30/Fc chimera, thus amplifying the inflammatory response.

In conclusion the study of CD30^+^ and CD30L^+^ cells helps in clarifying the complex pro- and anti-inflammatory mechanisms that take place in RA and possibly pave new avenues for the understanding of the response to therapy and envisage the possibility of novel treatments directed against the CD30L molecule.

## Figures and Tables

**Table 1:** CD30^+^ and CD30L^+^ T cells phenotype in the peripheral blood and synovial fluid of RA patients and controls.

RA patients (*n* = 14)	Sample	CD3^+^ CD4^+^	CD3^+^ CD8^+^	CD4^+^ CD30^+^	CD4^+^ CD30L^+^	CD8^+^ CD30^+^	CD8^+^ CD30L^+^	Treg	Treg CD30^+^
Mean ± S.D.	PB	73.9 ± 12.5	23.8 ± 12.3	0.8 ± 0.7	2.9 ± 1.8	0.1 ± 0.2	2.1 ± 0.2	5.0 ± 3.8	12.5 ± 15.2
Mean ± S.D.	SF	58.0 ± 10.7	38.8 ± 11.2	8.6 ± 5.6	13.9 ± 10.4	0.3 ± 0.5	2.3 ± 3.2	13.8 ± 4.8	42.3 ± 14.7

Controls (*n* = 8)	Sample	CD3^+^ CD4^+^	CD3^+^ CD8^+^	CD4^+^ CD30^+^	CD4^+^ CD30L^+^	CD8^+^ CD30^+^	CD8^+^ CD30L^+^	Treg	Treg CD30^+^

Mean ± S.D.	PB	72.3 ± 9.5	22.3 ± 10.2	0.3 ± 0.5	3.2 ± 0.9	0.3 ± 0.5	1.7 ± 1.7	4.5 ± 1.8	4.1 ± 4.3
Mean ± S.D.	SF	62.0 ± 7.1	33.0 ± 4.2	0.5 ± 0.7	10.0 ± 1.4	0.5 ± 0.7	6.9 ± 5.8	4.0 ± 2.8	19.0 ± 11.3

## References

[1] Smith C. A. (1993). CD30 antigen, a marker for Hodgkin's lymphoma, is a receptor whose ligand defines an emerging family of cytokines with homology to TNF. *Cell*.

[2] Gruss H.-J., Duyster J., Herrmann F. (1996). Structural and biological features of the TNF receptor and TNF ligand superfamilies: interactive signals in the pathobiology of Hodgkin's disease. *Annals of Oncology*.

[3] Berro A. I., Perry G. A., Agrawal D. K. (2004). Increased expression and activation of CD30 induce apoptosis in human blood eosinophils. *Journal of Immunology*.

[4] Agrawal B., Reddish M., Longenecker B. M. (1996). CD30 expression on human CD8+ T-cells isolated from peripheral blood lymphocytes of normal donors. *Journal of Immunology*.

[5] Romagnani S., Annunziato F., Manetti R. (1996). Role for CD30 in HIV expression. *Immunology Letters*.

[6] Gattei V., Degan M., Gloghini A. (1997). CD30 ligand is frequently expressed in human hematopoietic malignancies of myeloid and lymphoid origin. *Blood*.

[7] Gruss H.-J., Boiani N., Williams D. E., Armitage R. J., Smith C. A., Goodwin R. G. (1994). Pleiotropic effects of the CD30 ligand on CD30-expressing cells and lymphoma cell lines. *Blood*.

[8] Gruss H.-J., Dasilva N., Hu Z.-B., Uphoff C. C., Goodwin R. G., Drexler H. G. (1994). Expression and regulation of CD30 ligand and CD30 in human leukemia-lymphoma cell lines. *Leukemia*.

[9] Nicod L. P., Isler P. (1997). Alveolar macrophages in sarcoidosiscoexpress high levels of CD86 (B7.2), CD40 and CD30L. *American Journal of Respiratory Cell and Molecular Biology*.

[10] Pinto A., Aldinucci D., Gloghini A. (1996). Human eosinophils express functional CD30 ligand and stimulate proliferation of a Hodgkin's disease cell line. *Blood*.

[11] Shanebeck K. D., Maliszewski C. R., Kennedy M. K. (1995). Regulation of murine B cell growth and differentiation by CD30 ligand. *European Journal of Immunology*.

[12] Wiley S. R., Goodwin R. G., Smith C. A. (1996). Reverse signaling via CD30 ligand. *Journal of Immunology*.

[13] Younes A., Consoli U., Zhao S. (1996). CD30 ligand is expressed on resting normal and malignant human B lymphocytes. *British Journal of Haematology*.

[14] Molin D., Fischer M., Xiang Z. (2001). Mast cells express functional CD30 ligand and are the predominant CD30L-positive cells in Hodgkin's disease. *British Journal of Haematology*.

[15] Tinazzi E., Barbieri A., Rigo A. (2014). In rheumatoid arthritis soluble CD30 ligand is present at high levels and induces apoptosis of CD30^+^T cells. *Immunology Letters*.

[16] del Prete G., de Carli M., Almerigogna F. (1995). Preferential expression of CD30 by human CD4^+^ T cells producing Th2-type cytokines. *The FASEB Journal*.

[17] Gerli R., Lunardi C., Vinante F., Bistoni O., Pizzolo G., Pitzalis C. (2001). Role of CD30+ T cells in rheumatoid arthritis: a counter-regulatory paradigm for Th1-driven diseases. *Trends in Immunology*.

[18] Bengtsson A. (2001). The role of CD30 in atopic disease. *Allergy*.

[19] Polte T., Behrendt A.-K., Hansen G. (2006). Direct evidence for a critical role of CD30 in the development of allergic asthma. *Journal of Allergy and Clinical Immunology*.

[20] Saraiva M., Smith P., Fallon P. G., Alcami A. (2002). Inhibition of type I cytokine-mediated inflammation by a soluble CD30 homologue encoded by ectromelia (mousepox) virus. *The Journal of Experimental Medicine*.

[21] Tang C., Yamada H., Shibata K. (2008). A novel role of CD30L/CD30 signaling by T-T cell interaction in Th1 response against mycobacterial infection. *The Journal of Immunology*.

[22] Guo Y., Sun X., Shibata K. (2013). CD30 is required for activation of a unique subset of interleukin- 17A-Producing *γδ*T Cells in innate immunity against *Mycobacterium bovis* bacillus calmette-guérin infection. *Infection and Immunity*.

[23] Lin C.-Y., Graca L., Cobbold S. P., Waldmann H. (2002). Dominant transplantation tolerance impairs CD8^+^ T cell function but not expansion. *Nature Immunology*.

[24] Dai Z., Li Q., Wang Y. (2004). CD4^+^CD25^+^ regulatory T cells suppress allograft rejection mediated by memory CD8^+^ T cells via a CD30-dependent mechanism. *The Journal of Clinical Investigation*.

[25] Gaspal F. M. C., Kim M.-Y., McConnell F. M., Raykundalia C., Bekiaris V., Lane P. J. L. (2005). Mice deficient in OX40 and CD30 signals lack memory antibody responses because of deficient CD4 T cell memory. *The Journal of Immunology*.

[26] Zeiser R., Nguyen V. H., Hou J.-Z. (2007). Early CD30 signaling is critical for adoptively transferred CD4^+^CD25^+^ regulatory T cells in prevention of acute graft-versus-host disease. *Blood*.

[27] Gerli R., Muscat C., Bistoni O. (1995). High levels of the soluble form of CD30 molecule in rheumatoid arthritis (RA) are expression of CD30^+^ T cell involvement in the inflamed joints. *Clinical and Experimental Immunology*.

[28] Gerli R., Pitzalis C., Bistoni O. (2000). CD30^+^ T cells in rheumatoid synovitis: mechanisms of recruitment and functional role. *Journal of Immunology*.

[29] Gerli R., Bistoni O., Lunardi C. (1999). Soluble CD30 in early rheumatoid arthritis as a predictor of good response to second-line therapy. *Rheumatology*.

[30] Gerli R., Lunardi C., Bocci E. B. (2008). Anti-tumor necrosis factor-alpha response in rheumatoid arthritis is associated with an increase in serum soluble CD30. *The Journal of Rheumatology*.

[31] Miossec P. (2000). Are T cells in rheumatoid synovium aggressors or bystanders?. *Current Opinion in Rheumatology*.

[32] Aarvak T. T., Chabaud M., Källberg E., Miossecdagger P., Natvig J. B. (1999). Change in the Th1/Th2 phenotype of memory T-cell clones from rheumatoid arthritis synovium. *Scandinavian Journal of Immunology*.

[33] Nakamura T., Lee R. K., Nam S. Y. (1997). Reciprocal Regulation of CD30 Expression on CD4^+^ T Cells by IL-4 and IFN-*γ*. *The Journal of Immunology*.

[34] Pellegrini P., Berghella A. M., Contasta I., Adorno D. (2003). CD30 antigen: not a physiological marker for TH2 cells but an important costimulator molecule in the regulation of the balance between TH1/TH2 response. *Transplant Immunology*.

[35] Sun X., Somada S., Shibata K. (2008). A critical role of CD30 ligand/CD30 in controlling inflammatory bowel disease in mice. *Gastroenterology*.

[36] Sun X., Yamada H., Shibata K. (2010). CD30 ligand/CD30 plays a critical role in Th17 differentiation in mice. *Journal of Immunology*.

[37] Aletaha D., Neogi T., Silman A. J. (2010). 2010 Rheumatoid arthritis classification criteria: an American College of Rheumatology/European League Against Rheumatism collaborative initiative. *Arthritis & Rheumatology*.

